# Impact of MTHFR gene polymorphism on the outcome of methotrexate treatment in a sample of Iraqi rheumatoid arthritis patients

**DOI:** 10.1038/s41598-024-65199-7

**Published:** 2024-07-02

**Authors:** Qassim Mahdi Mutlak, Ali Abdulhussain Kasim

**Affiliations:** 1 Department of Clinical Pharmacy, College of Pharmacy, University of Baghdad, Baghdad, Iraq; 2 Department of Biochemistry and Clinical Laboratory Science, College of Pharmacy, University of Baghdad, Baghdad, Iraq

**Keywords:** Pharmacogenetic, Polymorphism, MTHFR, Methotrexate, Rheumatoid arthritis, rs1801133, rs1801131, Biochemistry, Rheumatic diseases, Rheumatoid arthritis

## Abstract

Analyze the relationship between genetic variations in the MTHFR gene at SNPs (rs1801131 and rs1801133) and the therapy outcomes for Iraqi patients with rheumatoid arthritis (RA). The study was conducted on a cohort of 95 RA Iraqi patients. Based on their treatment response, the cohort was divided into two groups: the responder (47 patients) and the nonresponder (48 patients), identified after at least three months of methotrexate (MTX) treatment. A polymerase chain reaction-restriction fragment length polymorphism (PCR–RFLP) technique was employed to analyze the MTHFR variations, specifically at rs1801133 and rs1801131. Overall, rs1801131 followed both codominant and dominate models, in which in the codominant model, GG [OR (95% CI) 0.11 (0.022–0.553)] and TG [OR (95% CI) 0.106 (0.021–0.528)] predict responders compared to the TT genotype; meanwhile, for the dominate model, the presence of both GG and TG genotypes [OR (95% CI) 0.108 (0.023–0.507)] together predict responders compared to the TT genotype. The A_rs1801133_G_rs1801131_ haplotype was significantly associated with responders [OR (95% CI): 0.388 (0.208–0.723)], while the G_rs1801133_T_rs1801131_ haplotype was associated marginally with nonresponders [OR (95% CI) 1.980 (0.965–4.064)]. In the final multivariate analysis, GG/TG_rs1801131_ genotypes were independently related to responders after adjustment for patients, disease, and treatment characteristics, while TT_rs1801131_ genotypes were associated with nonresponders. The Iraqi RA patients showed genetic polymorphism in MTHFR gene rs1801131 with T carrier allele associated with nonresponders to MTX therapy. The rs1801131 followed both codominant and dominant models. The G-carried allele for rs1801131 showed an independent association with responder to MTX therapy after adjustment for patients, disease, and treatment characteristics.

## Introduction

Rheumatoid arthritis (RA) is a persistent and widespread inflammatory condition that causes pain and swelling in joints on both sides of the body^1^. Insufficient management of chronic inflammation can lead to irreversible damage to the joint tissues^2^. Methotrexate (MTX) acts as a folic acid inhibitor, exhibiting anti-inflammatory and anti-proliferative activities by closely resembling folic acid concerning its structure and chemical properties. MTX is widely employed in the management of RA due to its substantial effectiveness, excellent safety record, and cost efficiency^3,4^. Although MTX is commonly used to treat RA, a substantial percentage of patients, ranging from 30 to 50%, do not respond well to MTX medication or experience a recurrence with inadequate response to re-treatment. This event results in the emergence of drug resistance, requiring either the cessation of MTX or the implementation of alternate pharmacological treatments^5^. The changes in gene expression or activity within the folic acid-MTX metabolic pathway may explain the variability in drug pharmacokinetics and response to MTX observed between patients^6^.

Folate metabolism is linked to the enzyme methylenetetrahydrofolate reductase (MTHFR). Methionine-methylation of homocysteine (Hcy) is made possible by the MTHFR enzyme, which catalyzes the conversion of 5,10-methylenetetrahydrofolate (5,10-CH2-THF) to 5-methyltetrahydrofolate (5-MTHF)^7^. This enzyme's regulatory genes have been intensively researched because of their importance in folic acid metabolism^7^.

Carrying heterozygous or homozygous variants of MTHFR polymorphisms is linked to a genetically set inclination towards higher levels of homocysteine in the blood^8^. These individuals are frequently diagnosed with mild hyperhomocysteinemia, with fasting plasma levels typically below 30 µmol/l in cases of dietary folate insufficiency^8^. The reduction in MTHFR activity is particularly noticeable in individuals who have both the rs1801133 variant in one allele of the MTHFR gene and the rs1801131 variant in the other allele^9^.

Folic acid is essential for preventing chromosomal breakage and hypomethylation of DNA. The activity is hindered when there is a low concentration of Vitamin B12 (B12) because it reduces the activity of methionine synthase, lowering the concentration of S-adenosyl methionine (SAM). This reduction in SAM may lead to a decrease in DNA methylation and prevent folate from being available for the conversion of dUMP to dTMP^10^.

The presence of a mutation in the MTHFR gene (rs1801133) can have a protective effect against cancer. However, it can also elevate the chance of developmental abnormalities, such as Down syndrome and neural tube anomalies^11–13^. The MTHFR mutation is likely to reduce the inclusion of uracil in DNA, thereby minimizing chromosome breakage and rearrangement. However, its effect on DNA methylation is relatively minor. This suggests that chromosome breakage and rearrangement may be more important than hypomethylation in cancer development. However, the significance of hypomethylation may vary depending on the intake of folate and the extent to which it causes aneuploidy, a potential carcinogenic event^14,15^. Nevertheless, the MTHFR mutation's influence on DNA methylation status could be significant throughout the intricately regulated development process, where the coordinated and timely expression of genes is crucial and potentially more sensitive to the correct DNA methylation status. The observations regarding the rs1801133 and rs1801131 MTHFR polymorphism demonstrate the potential significance of utilizing folate supplements to address metabolic constraints^16,17^. It is anticipated that a similar approach will apply to Vitamin B12 for deficiencies in other crucial enzymes like methionine synthase and methionine synthase reductase^18^. The presence of a mutation in the MTHFR gene (rs1801133) can have a protective effect against cancer. However, it can also elevate the chance of developmental abnormalities, such as Down syndrome and neural tube anomalies. The MTHFR mutation is likely to reduce the inclusion of uracil in DNA, thereby minimizing chromosome breakage and rearrangement. However, its effect on DNA methylation is relatively minor. This suggests that chromosome breakage and rearrangement may be more important than hypomethylation in cancer development. However, the significance of hypomethylation may vary depending on the intake of folate and the extent to which it causes aneuploidy, a potential carcinogenic event. Nevertheless, the MTHFR mutation's influence on DNA methylation status could be significant throughout the intricately regulated development process, where the coordinated and timely expression of genes is crucial and potentially more sensitive to the correct DNA methylation status. The observations regarding the C677T and A1298C MTHFR polymorphism demonstrate the potential significance of utilizing folate supplements to address metabolic constraints. A similar approach is expected to be applicable to Vitamin B12 for deficiencies in other crucial enzymes like methionine synthase and methionine synthase reductase^10^.

The enzyme expressed by the MTHFR gene on chromosome 1p36.3 plays an important role in cellular metabolism. Several polymorphic locations in the MTHFR gene have been found in previous research, with the SNP sites rs1801133 and rs1801131 being the most often investigated. Mutation in rs1801131 is linked to disruption of MTHFR enzymatic activities, which affect MTX activities in RA patients; however, not all ethnic groups have been tested to confirm this disruption^19–21^. Neither homozygotes nor heterozygotes for rs1801131 were associated with alteration in folic acid levels^22^. Investigating the link between MTX treatment response and MTHFR rs1801131 and rs1801133 polymorphisms yielded highly varied findings, leaving this pivotal connection doubtful. There is a lack of studies examining the effect of MTHFR genetic polymorphism and its consequence on MTX treatment response in Iraqi RA patients, which was the main drive behind this study.

The current study evaluates the association between genetic mutations in the MTHFR gene in rs1801133 and rs1801131 on the therapeutic outcomes for RA Iraqi patients, which has been reported previously in other nationality, and this is the first study to examine Arabic Iraqi RA patients with these polymorphisms.

## Methods

### Study design

A study was conducted using a sample of 95 patients from Iraq who had been diagnosed with rheumatoid arthritis (RA) based on the revised 2010 criteria set by the American College of Rheumatology (ACR) and the European League Against Rheumatism (EULAR) for the classification of RA^23^.

Patients were divided into two groups according to a previous study^24^, in which the responder group was defined as DAS28 ≤ 3.2, and the nonresponder DAS28 > 3.2 after at least three months of MTX treatment^24–26^. Responder groups included 48 patients and 47 patients as nonresponders.

The study was prepared per STREGA guidelines for reporting genetic association studies^27^.

### Study settings

The participants in this study were selected from the Rheumatology Department of Diwaniya Teaching Hospital. The study was conducted from June 1, 2022, to March 1, 2023.

### Inclusion criteria

Adult patients (age ≥ 18 years) with confirmed RA according to revised 2010 ACR/EULAR RA classification criteria^23^, and all patients should be on MTX for at least three months.

### Exclusion criteria

Patients with comorbidities, concurrent connective tissue diseases, patients on concomitant disease-modifying anti-rheumatic drugs (DMARDs) or biological drugs, incomplete data, patients with chronic infectious diseases, cancer, hepatic or renal impairment, endocrinopathy, hematological disorders, and cardiac conditions.

### Variables

#### Disease activity (DAS28-ESR)

In the present study, DAS28-ESR (four variables) was used to examine the disease activity of RA patients^28^. This score is a validated method for such purpose and has validated many studies and recognized by ACR/EULAR^29–32^, and it was calculated based on the following equation^28^:

$$DAS28-4=0.56*\surd (\text{Tender \, Joint \, Count}-28) + 0.28*\surd (\text{Swollen \,  Joint \ Count}-28) + 0.70*ln(ESR) + 0.014*(General \, Health)$$

Patients were classified as remission (< 2.6), low (≥ 2.6 – ≤ 3.2), moderate (> 3.2 – ≤ 5.1), and high (> 5.1)^32^.

#### Laboratory analysis

Measurement of erythrocyte sedimentation rate (ESR) was conducted using the modified Westergren method, which involves the use of whole blood that has been anticoagulated with EDTA (VISION Pro ESR Analyzer, Shenzhen YHLO Biotech Co., Ltd)^33–35^, while rheumatoid factor **(**RF**)** was measured using a Chemistry Analyzer, Smart—150, Geno Lab-TEK Corporation, Canada.

#### Disease response

Patients were divided into two groups according to a previous study^24^, in which the responder group was defined as DAS28 ≤ 3.2, and nonresponder DAS28 > 3.2 after three months of MTX treatment^24–26^.

#### Sample size

Power analysis was used to calculate the sample size, G*Power version (3.1.9.7)^36,37^. The total sample size was 97, with an alpha probability of 0.05, power of 88%, and one-tailed using one sample case variance.

#### DNA extraction

**Figure 1:**
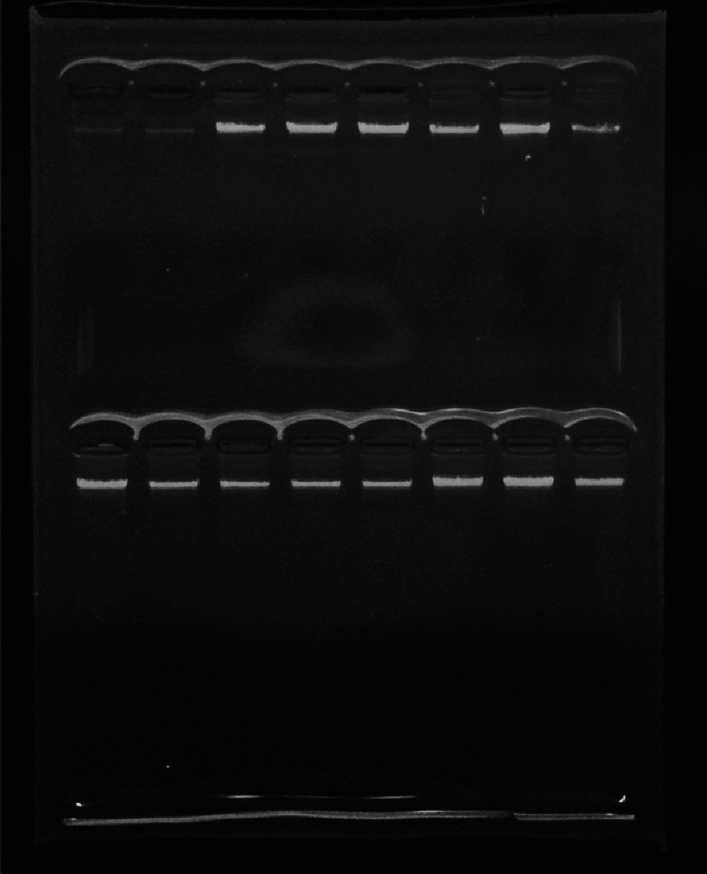
Extracted genomic DNA electrophoresis on 1% agarose.

The genomic DNA was extracted from 5 ml of venous blood obtained from the individuals and subsequently stored at a temperature of − 20 °C until further analysis. The isolation of DNA relies on inorganic methods^38^. Following agarose gel electrophoresis (1% agarose) using the Bio-Rad Experion Automated Electrophoresis System (RRID: SCR_019691), all images were analyzed using the CS analyzer v3 software by ATTO, Japan. The DNA samples were then validated using spectrophotometry with the Shimadzu UV-1800 UV/Visible Scanning Spectrophotometer; 115 VAC (Germany)^39,40^, see Fig. 1.

#### Primer selection

The process of designing PCR primers for MTHFR variants (rs1801133 and rs1801131) is outlined in Table 1. Here is a brief summary: The primers were designed using the NCBI-primer BLAST online software [NCBI BLAST (RRID: SCR_004870)]. The specificity of the produced primers for their target sequences was confirmed by performing a BLAST against the human genome. The primers pair was then selected based on criteria such as product length, melting temperature similarity, primers length, and specificity. The primer's capacity to generate a secondary structure was assessed using the Oligo Calc online software [OligoCalc (RRID: SCR_022663)]. The primer would be deemed unsuitable if it included five or more bases capable of forming self-dimerization or if it had four bases capable of creating a hairpin structure. The generation of primer dimers was assessed for each primer pair using the "Multiple Primer Analyzer" online software provided by Thermo Fisher Scientific Inc.©. The software's sensitivity was set to a value of 2, and any primer pair that produced dimers at this level of sensitivity was deemed unsuitable and excluded^41–43^.

**Table 1:** Primers sequence, GC%, annealing temperature (Ta), and product size of *MTHFR* genes 1298 (rs1801131) SNP and 677 (rs1801133) SNP (302).

*MTHFR* gene 1298 (rs1801131) SNP		
Ta	60 °C	
Product size	90 bp	
**Primers**	**Sequence**	**GC%**
Forward primer	TCCCGAGAGGTAAAGAACGtAGAC	50
Reverse primer	TCCCCCAAGGAGGAGCTGCTGtAGA	60

*MTHFR* Gene 677 (rs1801133) SNP		
Ta	60 °C	
Product size	254 bp	
**Primers**	**Sequence**	**GC%**
Forward primer	CCTGGATGGGAAAGATCCCG	60
Reverse primer	CATCCCTCGCCTTGAACAGG	60

*Ta* annealing temperature

#### Genotyping of polymorphisms

The MTHFR variations (rs1801133 and rs1801131) were analyzed using a polymerase chain reaction-restriction fragment length polymorphism (PCR–RFLP) technique. The Macrogen Company from Korea supplied the forward and reverse primers in lyophilized state. All polymerase chain reaction (PCR) processes need the use of a PCR thermal cycler, which is manufactured in Germany. The reactions were modified to a total volume of 20 µl, including of Master mix probes and 10–20 ng/µl of genomic DNA (refer to Table 2). The amplification technique is outlined in Table 3.

**Table 2:** The Components of PCR for Amplification *MTHFR* Gene.

Reagents	Concentration	Volume
Genomic template DNA	10–20 ng/μl	2 μl
Master mix	2.5 ×	8 μl
Forward primer (100 pmol/µl)	10 pmol/μl	1 μl
Reverse primer (100 pmol/µl)	10 pmol/μl	1 μl
MgCl_2_	25 mM/0.5 ml	0.5 μl
Nuclease free water	–	7.5 μl
reaction total volume	–	20 μl

**Table 3:** The PCR protocol for Amplification of *MTHFR* Gene.

Stage	Step	Temperature	Interval	Cycles number
1	Initial denaturation	94 °C	5 min	1
2	Denaturation	94 °C	30 s	35
3	Annealing	60 °C	30 s	
4	Elongation	72 °C	30 s	
5	Final elongation	72 °C	5 min	1

#### Restriction fragment length polymorphism analysis for MTHFR rs1801133‏ and rs1801131 genotyping

Restriction fragment length polymorphism (RFLP) analysis was performed; the basic principle of RFLP involves the fragmentation of a DNA sample via a specific restriction enzyme that acts on a specific restriction site, producing different fragment lengths for different variants due to loss or gain of a restriction site by mutations. The fragments with different lengths of digested DNA are separated using gel electrophoresis depending on the size of these fragments^44^.

Suitable restriction enzymes (Table 4) were selected using SnapGene viewer software (V6.0.5).‏ ‏The reagents and conditions utilized for the restriction reactions are presented in Table 5. The reaction mixtures were incubated overnight at 37 °C in a water bath for complete digestion by the restriction enzyme.

**Table 4:** The restriction enzymes used in the study.

Enzyme	SNP	Recognition sequence	Supplier
Mbo II	rs1801131	GAAGA(N)2↓ CTTCT (N)2↑	SibEnzyme (Russia)
PspN4I	rs1801133	GGN↓NCC CCN↑NGG	SibEnzyme (Russia)

**Table 5:** Reagents and conditions utilized in MTHFR rs1801133 and rs1801131 restriction reaction.

rs1801133		rs1801131	
Reagents‏ ‏ and conditions	Volumes	Reagents‏ ‏ and conditions	Volumes
Water free of nuclease	8 μl	Water free of nuclease	8.7 μl
Enzyme buffer 10 ×	1.5 μl	Enzyme buffer 10 ×	1.5 μl
Yield of PCR	5 μl	Yield of PCR	5 μl
Restriction enzyme (PspN4I)	0.5 μl	Restriction enzyme (Mbo II)	0.3 μl
Overall volume	15 μl	Overall volume	15 μl
Period of incubation	Overnight	Period of incubation	Overnight
Temperature of incubation	37 °C	Temperature of incubation	37 °C

#### Electrophoresis of the digested products

The digestion yields of rs1801133 were assessed utilizing electrophoresis via running 5 μl of each sample in 2% agarose gel, while these of rs1801131 using 8% polyacrylamide gel and ethidium bromide nucleic acid staining solution. Applying a 100-V current for 1 h separated the digestion products. The electrophoretic bands were later visualized through a U.V. transilluminator for agarose gel. The electrophoretic bands in polyacrylamide gel were detected using white light. The determination of genotypes was based on the band patterns.

**Figure 2:**
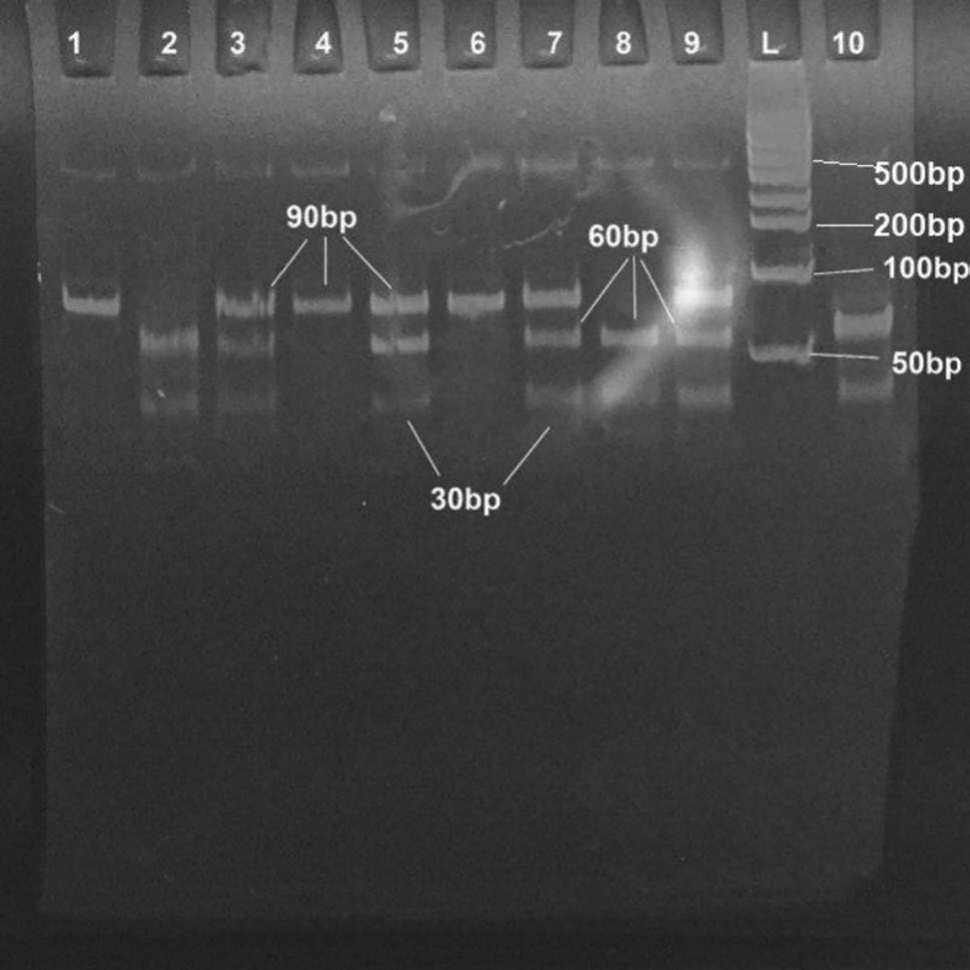
Genotyping of rs1801131 polymorphism by PCR–RFLP technique, the PCR product (90 bp) was digested with MboI restriction enzyme, T allele would be recognized by restriction enzyme and cut the amplicon to produce 30 and 60 bp fragment, on the other hand, the G allele did not recognize by the restriction enzyme and produce the original 90 bp fragment. Genotype pattern: TT genotype (30 and 60 bp fragments) as shown in lanes 2, 8, and 10; GG genotype pattern (90 bp fragment) as shown in lanes 1, 4 and 6; TG genotype (30, 60, and 90 bp fragments) as shown in lanes 3, 5, 7 and 9. lane marked with L: 100 + 50 DNA ladder. The product restriction product was resolved on a polyacrylamide gel. [Bio-Rad Experion Automated Electrophoresis System (RRID: SCR_019691)].

The amplification of the MTHFR gene exhibited an amplicon of 90 bp that contains the target SNP rs1801133. The product digestion by a restriction enzyme revealed three bands’ patterns. The product digestion by a restriction enzyme explored three patterns. One uncut fragment (90 bp) band represents the wild genotype (GG). In contrast, two bands (30 bp) and (60 bp), represent the homozygous genotype (TT), and three bands (30 bp), (60 bp), and (90 bp) represent the heterozygous genotype (TG), as seen in Fig. 2.

**Figure 3:**
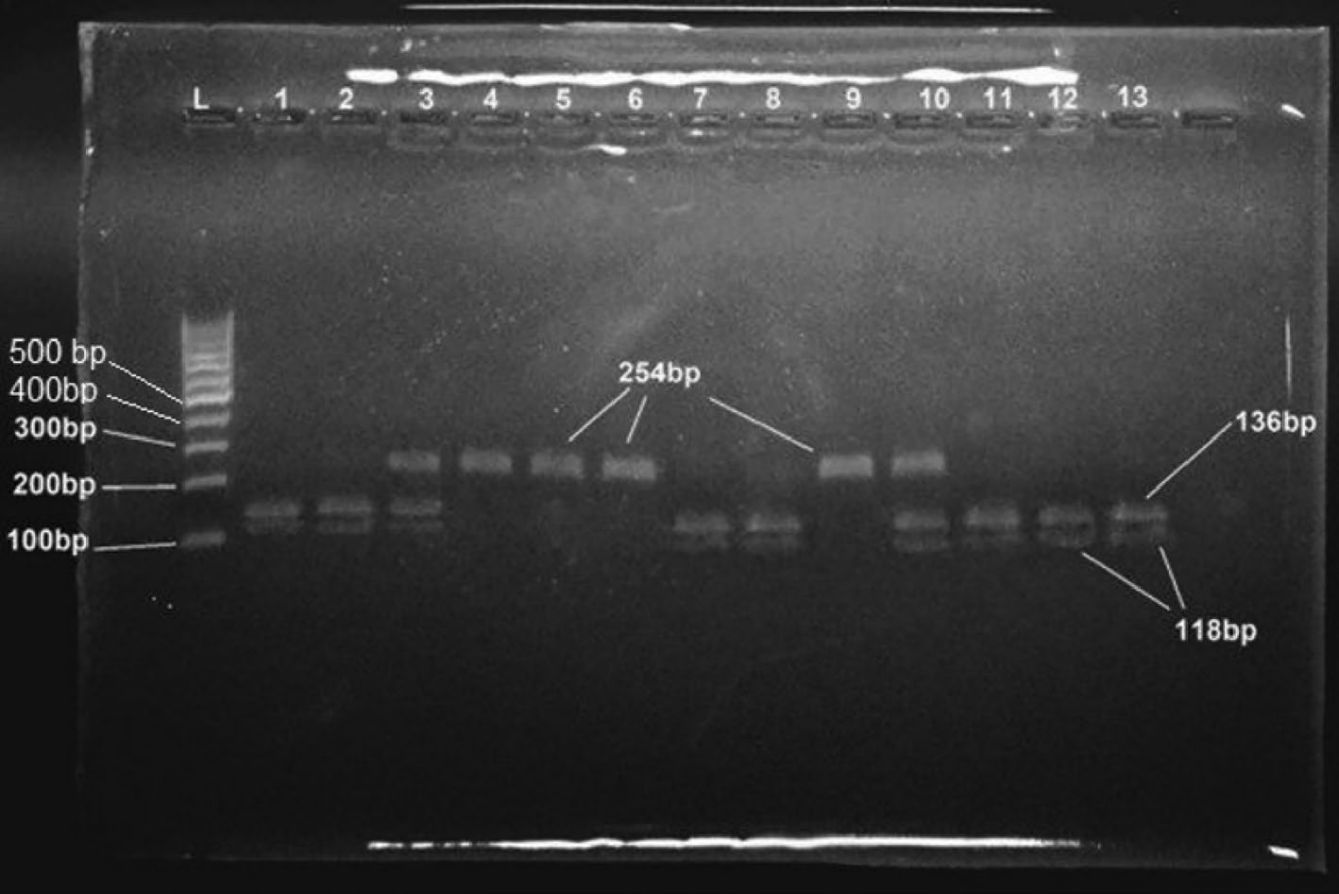
Genotyping of rs1801133 polymorphism by PCR–RFLP technique, the PCR product (254 bp) was digested with PspN4I restriction enzyme, G allele would be recognized by restriction enzyme and cut the amplicon to produce 118 and 136 bp fragments, on the other hand, the G allele did not recognize by the restriction enzyme and produce the original 254 bp fragment. Genotype pattern: GG genotype (118 and 136 bp fragments) as shown in lanes 1, 2, 7, 8, 11, 12, and 13. AA genotype pattern (254 bp fragment) as shown in lanes 4, 5, 6, and 9. AG genotype (118, 136, and 254 bp fragments) as shown in lanes 3 and 10. The lane was marked with an L: 100 bp DNA ladder. The restriction product was resolved on 2% agarose gel. [Bio-Rad Experion Automated Electrophoresis System (RRID: SCR_019691)].

The amplification of the MTHFR gene exhibited an amplicon of 254 bp that contains the target SNP rs1801133. The product digestion by a restriction enzyme revealed three band patterns. One uncut fragment (254 bp) band represents the wild genotype (AA). In contrast, two bands (118 bp) and (136 bp) represent the homozygous genotype (GG), and three bands (254 bp), (118 bp) and (136 bp) represent the heterozygous genotype (AG), as seen in Fig. 3.

### Ethics approval

The study was approved by "The University of Baghdad-College of Pharmacy Research Ethics Committee" (Approval number: RECAUBCP 6620226, date: 6th June 2022), and written informed consent was obtained from all participants in the study, in accordance with the Helsinki Declaration and its later amendments.

### Statistical analysis

An analysis was conducted on the genotyping results to calculate the frequency of alleles and genotypes. The Hardy–Weinberg Equilibrium (HWE) Calculator for 2 Alleles is an online tool that calculates the difference in distribution between the observed frequency of genotypes and the expected frequency of genotypes^45^. Both rs1801133 (p-value = 0.018) and rs1801131 (p-value = 0.304) adhere to Hardy–Weinberg Equilibrium (HWE) with a p-value of 0.0057 used for allele frequency less than 100, assuming a type I error (α) of 0.01^46^.

The analysis of haplotypes was conducted using the SHEsis online software, which utilizes the partition-ligation-combination-subdivision EM algorithm for inferring haplotypes using markers that have multiple alleles^47,48^.

The Shapiro–Wilk test is used to evaluate the adherence of variables to a normal distribution. The chi-square test is employed to ascertain categorical variables. The Fisher exact test is employed as an alternative to the chi-square test in situations when the sample size is below 20 or when there are two or more categories with anticipated frequencies below 5, as the chi-square test is not suitable in such scenarios. The Fisher–Freeman–Halton exact test of independence is used for n x k tables, which is an expansion of the traditional 2 × 2 Fisher exact test where the expected frequency is less than 5%^49^. The study utilized independent t-tests to analyze the differences in means between the two groups, assuming that both groups follow a normal distribution and do not include any significant outliers. The Mann–Whitney *U* test is a non-parametric statistical test utilized to assess whether there is a significant difference between two independent groups. The *U* test was utilized to assess the differences in the median values between the two groups, especially when the data does not follow a normal distribution^50^. The study utilized binary logistic regression analysis to calculate the odds ratio (OR) and their related 95% confidence intervals. This statistical method is appropriate for cases when the result variable may be categorized into two binary levels.

In addition, the Wald test, which conforms to a Chi-Square distribution with one degree of freedom, was employed to assess the relative magnitude of the parameters. In the framework of multivariate binary logistic regression analysis, an unconditional model was used to investigate the different factors related to patients (model 1), disease (model 2), and therapy (model 3). In addition, the possible interaction between different single nucleotide polymorphisms (SNPs) was taken into account by using multivariate analysis. The statistical analysis was conducted using GraphPad Prism version 10.0.0 for Windows [GraphPad Prism (RRID: SCR_002798)]. A p-value less than 0.05 was considered significant when acceptable.

### Consent to participate

Written informed consent obtained.

## Results

The study comprised 95 patients diagnosed with rheumatoid arthritis (RA), with an average age of 43.1 ± 10.6 years. The majority of the patients were female (85.3%), approximately 35.8% were smokers. Among the patients, 45.2% had low disease activity, followed by 41.1% with moderate activity, 9.5% with high activity, and 4.2% in remission. Out of the total, 49.5% of the participants were classified as responders, which means their Disease Activity Score (DAS28) was less than or equal to 3.2 after three months of treatment with MTX. The other participants, whose DAS28 was greater than 3.2, were classified as nonresponders^24–26^.

There was no significant difference in age, sex, and smoking; meanwhile, ESR, RF, DAS28, MTX dose and duration, duration of disease, and side effect (SE) were significantly lower in responders compared to nonresponders, as illustrated by Table 6.

**Table 6:** Assessment of demographic, laboratory, and disease characteristics of RA patients classified by response.

Variables	Responder	Nonresponder	p-value
Number	47	48	–
Age (years), mean ± SD	42.7 ± 10.2	43.4 ± 11.1	0.759
Sex, n (%)			0.966
Female	40(85.1%)	41(85.4%)	
Male	7(14.9%)	7(14.6%)	
Smoking, n (%)	20(42.6%)	14(29.2%)	0.174
ESR (mm/h), median (IQR)	18 (14–22)	38.5 (25–53.8)	< 0.001 [S]
RF (U/ml), median (IQR)	18 (14.9–23)	28.5 (23.5–37.4)	< 0.001 [S]
DAS28 score, median (IQR)	2.8 (2.7–3)	3.9 (3.5–5.0)	< 0.001 [S]
MTX dose (mg), median (IQR)	7.5 (7.5– 10)	10 (7.5–10)	0.005 [S]
MTX duration (month), median (IQR)	12 (9–24)	28 (11–41.5)	0.003 [S]
Duration of disease, median (IQR)	19 (9–29)	30 (16.3–46.8)	0.004 [S]
SE, n (%)	38(80.9%)	46(95.8%)	0.023 [S]

*IQR* interquartile range, *n* number, *ESR* erythrocyte sedimentation rate, *RF* rheumatoid factor, *DAS* disease activity score, *SE* side effect, *S* Significant.

Genotyping was done for all RA patients (95) with no genotyping failure. For both SNPs, the results are reported in electrophoresis see Figs. 2 and 3.

Three genetic models were taken into consideration during the analyses to determine the relationship between genetic polymorphism and RA disease activity:

- a codominant (three distinct genotypes for rs1801131: TT, TG, and GG. and for rs1801133: GG, AG, and AA),
- a dominant (For the minor allele, heterozygotes and homozygotes were paired together for rs1801131: GG plus TG vs. TT. And for rs1801133: AA plus AG vs. GG.)
- recessive model (GG vs. TG plus TT are homozygotes for the minor allele for rs1801131. And for rs1801133: AA vs AG plus GG).

The distribution of genotypes for the rs1801131 SNP was significantly different among the three genotypes. For the single alleles, the TT allele showed the highest frequency in nonresponders, and the T allele showed the highest frequency in nonresponders. Meanwhile, the genotype distribution for the (rs1801133) SNP was not significantly different among the three genotypes and for the single alleles, as illustrated by Table 7.

**Table 7:** Assessment of genotype of MTHFR gene (rs1801131T>G) and (rs1801133G>A) SNP and allele frequency according to disease activity.

	Responder	Nonresponder	p-value
Number	47	48	–
rs1801131T>G genotype			
GG	22 (46.8%)	17 (35.4%)	0.005
TG	23 (48.9%)	17 (35.4%)	
TT	2 (4.3%)	14 (29.2%)	
Allele			
G	67 (71.3%)	51 (53.1%)	0.010
T (wild)	27 (28.7%)	45 (46.9%)	
rs1801133G>A genotype			
AA	17 (36.2%)	13 (27.1%)	0.153
AG	20 (42.6%)	16 (33.3%)	
GG	10 (21.3%)	19 (39.6%)	
Allele			
A	54 (57.4%)	42 (43.8%)	0.059
G (wild)	40 (42.6%)	54 (56.3%)	
The chi-square test or Fisher exact test			

Overall, rs1801131 SNP followed both codominant and dominate models, in which in the codominant model, GG and TG predict responders compared to the TT genotype; meanwhile, for the dominate model, the presence of both GG and TG genotype together predict responders compared to the TT genotype. There was no significant difference in the distribution of three genotypes according to the three genetics models for rs1801133 SNP, as illustrated by Table 8.

**Table 8:** Assessment of the association between MTHFR gene (rs1801131T>G) SNP and (rs1801133G>A) SNP polymorphism and disease activity using the three genetic models to predict nonresponders.

Genotype	Responder	Nonresponder	OR (95%CI)	p-value
rs1801131 SNP				
Codominant model				
GG	22 (56.4%)	17 (43.6%)	0.11 (0.022–0.553)	0.007
TG	23 (57.5%)	17 (42.5%)	0.106 (0.021–0.528)	0.006
TT	2 (12.5%)	14 (87.5%)	–	–
Dominant genetic model				
GG+TG	45 (57.0%)	34 (43.0%)	0.108 (0.023–0.507)	0.005
TT	2 (12.5%)	14 (87.5%)	–	–
Recessive genetic model				
TT+TG	25 (44.6%)	31 (55.4%)	1.605 (0.704–3.656)	0.260
GG	22 (56.4%)	17 (43.6%)	–	–
rs1801133 SNP				
Codominant model				
AA	17 (56.7%)	13 (43.3%)	0.402 (0.140–1.153)	0.090
AG	20 (55.6%)	16 (44.4%)	0.421 (0.153–1.155)	0.093
GG	10 (34.5%)	19 (65.5%)	Reference	–
Dominant genetic model				
AG+AA	37 (56.1%)	29 (43.9%)	0.413 (0.167–1.022)	0.058
GG	10 (34.5%)	19 (65.5%)	–	–
Recessive genetic model				
GG+AG	30 (46.2%)	35 (53.8%)	1.526 (0.638–3.647)	0.342
AA	17 (56.7%)	13 (43.3%)	–	–
Binary logistic regression				

The AG haplotype was significantly associated with responders with OR: 0.388 (p-value = 0.003), while the GT haplotype was associated marginally with nonresponders OR: 1.98 (p-value = 0.060), as illustrated by Table 9.

**Table 9:** Haplotype analysis of the 2 SNPs with the overall response rate.

Haplotyping	Nonresponder	Responder	OR [95% CI]	p-value
A_rs1801133_G_rs1801131_	22.58 (0.235)	41.57 (0.442)	0.388 [0.208 ~ 0.723]	0.003
A_rs1801133_T_rs1801131_	19.42 (0.202)	12.43 (0.132)	1.664 [0.765 ~ 3.620]	0.196
G_rs1801133_G_rs1801131_	28.42 (0.296)	25.43 (0.271)	1.134 [0.603 ~ 2.133]	0.696
G_rs1801133_T_rs1801131_	25.58 (0.266)	14.57 (0.155)	1.980 [0.965 ~ 4.064]	0.060

Global result: Global χ^2^ is 10.32 while df = 3 (if frequency < 0.03 in both nonresponder & responder has been dropped). Fisher's p-value is 0.016.

*CI* confidence interval.

In the final multivariate analysis, GG/TG_rs1801131_ genotypes were independently associated with responders after adjustment for patients, disease, and treatment characteristics, while TT_rs1801131_ genotypes were related to nonresponders, as illustrated by Table 10.

**Table 10:** Multivariate analysis of different genotypes to predict nonresponder.

Genotype	Unadjusted		Model 1		Model 2		Model 3	
	OR (95% CI)	p-value	OR (95% CI)	p-value	OR (95% CI)	p-value	OR (95% CI)	p-value
rs1801133G>A								
AA	0.476 (0.158–1.435)	0.187	0.485 (0.156–1.504)	0.210	0.578 (0.171–1.957)	0.378	0.752 (0.194–2.918)	0.680
AG	0.435 (0.148–1.273)	0.128	0.432 (0.144–1.291)	0.133	0.340 (0.102–1.140)	0.080	0.344 (0.095–1.237)	0.102
GG	1.0	0.266	1.0	0.285	1	0.217	1	0.237
rs1801131T>G								
GG	0.114 (0.022–0.581)	0.009	0.102 (0.019–0.557)	0.008	0.090 (0.016–0.521)	0.007	0.051 (0.005–0.523)	0.012
TG	0.114 (0.023–0.581)	0.009	0.107 (0.020–0.568)	0.009	0.080 (0.014–0.466)	0.005	0.057 (0.006–0.582)	0.016
TT	1.0	0.025	1.0	0.023	1	0.016	1	0.039

Model 1 excludes the effect of age, sex, and smoking.

Model 2, in addition to variables in Model 1, excludes disease duration.

Model 3, in addition to variables in models 1 and 2, excludes MTX dose, duration, and NSAID.

## Discussion

The genetic role in predisposition, development, severity, and response for therapeutic intervention of RA is well documented, and MTX is one of the most intriguing subjects for pharmacogenomics research. Pharmacogenetic studies facilitate the discovery of response predictors for optimal efficacy with the least harm^43,51–55^.

In Iraq, the prevalence survey for rheumatoid arthritis was done during the summer of 1975 in persons aged 16 and over in areas of Iraq; definite Rheumatoid arthritis was observed in 1% of the population^56^. Additional studies reported prevalence as high as 1.93%^57^, and 2.34%^58^.

Regarding the demographic variables, the mean age of the patients was 43.1 ± 10.6 years, with females representing the majority of the patients (85.3%). In Iraq, the published literature showed similarities to observed demographic data in 470 RA patients in Basrah city, revealing a mean age of 49.9 ± 11.9 years, and 81.9% of the patients were female^59^. In another study involving 1039 RA patients in Babel city, 82.09% were female and had a mean age of 44.87 ± 3.63 years^60^. In a study in Erbil City, the mean age was 47.4 ± 10.6 years, with females representing 72.30% of the patients^61^. In a study done in Baghdad city, the mean age was 46.5 years, with 58.1% of the patients being females^62^. In a large study that involved several Iraqi cities, the mean age was 48.1 ± 12.1 years, and 83% were females^63^. The consistency of the current study regarding age and sex with the previous Iraqi study indicates the selection of RA patients in the current study is a good representative of all RA patients; thus, the current study finding can be generalized.

Pharmacogenomics studies concerning RA have emphasized the identification of genetic markers that may predict a patient's clinical response or the development of side effects for a given therapy, as well as the assessment of potential interactions between a patient's genetic profile and environmental factors^64–67^.

This field holds promise for advancing personalized medicine and enhancing RA treatment approaches. In the present study, the genetic association between MTHFR gene rs1801131 and rs1801133 SNP polymorphism and MTX response was sought; it was observed that the rs1801131 genotype follows both codominant and dominant genetic model, in which GG/TG_rs1801131_ genotype in codominant/dominate models predict responder to treatment, while TT_rs1801131_ genotype predict nonresponder. In the final multivariate analysis, the GG/TG_rs1801131_ genotypes were independently associated with responders after adjustment for patients, disease, and treatment characteristics, while TT_rs1801131_ genotypes were associated with nonresponders. This phenomenon could be attributed to decreased MTHFR activity, resulting in lower levels of 5-MTHF and other folate cofactors. This reduction may lead to a decrease in adenosine release, partly explaining the lack of response to MTX.

In haplotyping analysis, the AG haplotype for rs1801133–rs1801131 was found to be significantly associated with responders, while the GT haplotype for rs1801133–rs1801131 was associated marginally with nonresponders OR: 1.98 (p-value = 0.060).

This is the first study that examined the Arabic Iraqi population; however, recently, in another study that examined a different ethnic group (namely Kurdish) Iraqi RA patients, the authors concluded that genetic polymorphisms of MTHFR SNP (rs1801133 and rs1801131) are associated with MTX efficacy but not toxicity in RA patients^68^. The current study only found that rs1801131 was associated with treatment response, regarding the Kurdish-Iraqi study^68^, and in our study, no association between rs1801133 and rs1801131 with sex was found.

Similar to the current study, in Urano et al., the rs1801131 allele was associated with lower doses of MTX. At the same time, rs1801133 showed no significant difference between GG and TG allele (GG showed more association) and had a significantly higher association with good response (more reduction of ESR and CRP), while RS1801133 showed no association^69^. In haplotyping analysis, 677C–1298G was associated with lower doses of MTX^69^.

Another similar study reported that rs1801133 and rs1801131 alleles were associated with response in multivariate analysis; the following diplotypes CC/GG, TA/AG, CC/AG, CA/AG, and CA/GG were significantly associated with a lower probability of response compared to CA/GT (rs1801133–rs1801131)^70^. In the study conducted by Xiao et al., it was shown that the allele frequency of rs1801131G in the clinical response group was substantially greater compared to the non-response group (21.0% vs. 8.1%, p < 0.05). Furthermore, it was found that patients with the GG or TG genotype had a more pronounced clinical reaction than those with the TT genotype^71^.

Sharaki et al. examined 50 Arabic Egyptian patients with RA of similar age, sex, and disease duration. The authors read MTHFR SNP (rs1801131), in their multivariate analysis, found that MTHFR rs1801131 (TG + GG genotypes) independently associated with higher odds of non-response to MTX (OR 39.1, 95% CI 2.0–776.6), however they did not examine the effect of genetic polymorphism aside from MTHFR rs1801131^24^, which is to the contrary to the current study. In Algerian RA patients, no association was found between RS1801133 polymorphism and MTX efficiency. In contrast, the T allele of the T1298G polymorphism was associated with good and moderate response (p = 0.02, OR 3.28, 95% CI [1.11–9.42])^72^. It might be linked to a decline in MTHFR function, which also results in lower quantities of 5-MTHF and other folate cofactors, reducing adenosine releases and partially explaining MTX non-response^73,74^. Kato et al. studied 150 RA Japanese patients, and the mean age was more than 59 years, with similar DAS. The authors examined several SNPs, including the MTHFR genes rs1801131 and rs1801133. Contrary to the current study, the TT genotype for rs1801131 SNP had lower DAS28 compared to the TG/GG genotype (3.15 vs. 3.92, p-value = 0.04), which indicates a better response conferred by the T allele^75^, in Wessels et al. study that examined 205 RA Netherlands patients with older age and similar sex distribution. The authors examined the same MTHFR gene rs1801131 and rs1801133 SNP polymorphism; for rs1801133 SNP no the genetic polymorphism did not associate with MTX treatment response, while the TT genotype of rs1801131 was associated with a good response^76^, their findings are best applied to patients with early RA who have not received DMARDs. Van Ede et al. found no significant difference in the average change in DAS44 in MTHFR 677 T allele carriers versus noncarriers^77^. Research has demonstrated that the length of a condition and the prior treatment received have a significant impact on the result of methotrexate (MTX) treatment^78,79^, and their cohort included patients with persistent RA who had previously taken other DMARDs.

In Kumagai et al. study, MTHFR rs1801133 and rs1801131 polymorphisms showed no association with MTX-related efficacy^80^. A meta-analysis comprising ten studies found that the MTHFR gene RS1801131 polymorphism did not significantly impact the outcome of methotrexate (MTX) treatment in patients with rheumatoid arthritis (RA). However, subgroup analysis revealed a significant correlation between the MTHFR gene rs1801131 polymorphism and reduced efficacy of MTX in the South Asian population. Specifically, individuals with the GG genotype had a lower odds ratio (OR) of 0.45 (95% confidence interval [CI] 0.23–0.89, p = 0.021) compared to those with the GT + TT genotypes. Furthermore, the presence of the MTHFR gene rs1801131 polymorphism in the group receiving partial folate supplementation was found to be associated with a decrease in the effectiveness of MTX (GG vs. GT + TT: odds ratio = 0.43, 95% confidence interval 0.20–0.92, p = 0.029)^81^.

The reasons behind the higher prevalence of MTX and MTHFR response in female patientscould be explained by various theories, such as the hormonal differences between females and males and genetic factors that could be unique to the Iraqi Arabic female population.

### Study limitations

Reproducing findings in genetic association research is intrinsically intricate, resulting in difficulties in making significant comparisons across many studies. The strongest evidence of a correlation is in reproducing this association in a separate population that possesses the same genetic makeup, physical characteristics, and direction of influence^76^. The majority of research examining genetic connections suffer from insufficient statistical power as a result of the limited number of individuals with the homozygous mutant genotype^76^.

One of the challenges encountered when assessing the effectiveness of treatment in rheumatoid arthritis (RA) is the requirement for agreement on the measures used to determine efficacy. There exists a divergence of viewpoints regarding issues such as the duration and experimental approach required^82–87^. In therapeutic applications, it is desirable to establish definitive threshold values that can be used to categorize patients as either responders or nonresponders, hence facilitating the development of treatment protocols. Nevertheless, within clinical practice, clinicians are inclined to modify treatment regimens in response to any observed alteration in efficacy measurements^88,89^.

## Conclusion

The Iraqi RA patients showed genetic polymorphism in rs1801131 SNP with T carrier allele associated with nonresponder to MTX therapy; the rs1801131 SNP followed both codominant and dominate models. The G-carried allele for rs1801131 showed an independent association with responder to MTX therapy after adjustment for patients, disease, and treatment characteristics. While rs1801133 SNP did not associate with MTX responsiveness (Supplementary figures).

### Supplementary Information

Supplementary Figures.

## Data Availability

Zenodo: MTHFR gene polymorphism. 10.5281/zenodo.8392701. Data are available under the terms of the Creative Commons Attribution 4.0 International license (CC-BY 4.0).
