# Supplementary figures and images for: Impact of MTHFR gene polymorphism on the outcome of methotrexate treatment in a sample of Iraqi rheumatoid arthritis patients
Source: Sci Rep. 2024 Jul 2;14:15119. doi: 10.1038/s41598-024-65199-7 (PMC11219777; doi:10.1038/s41598-024-65199-7)

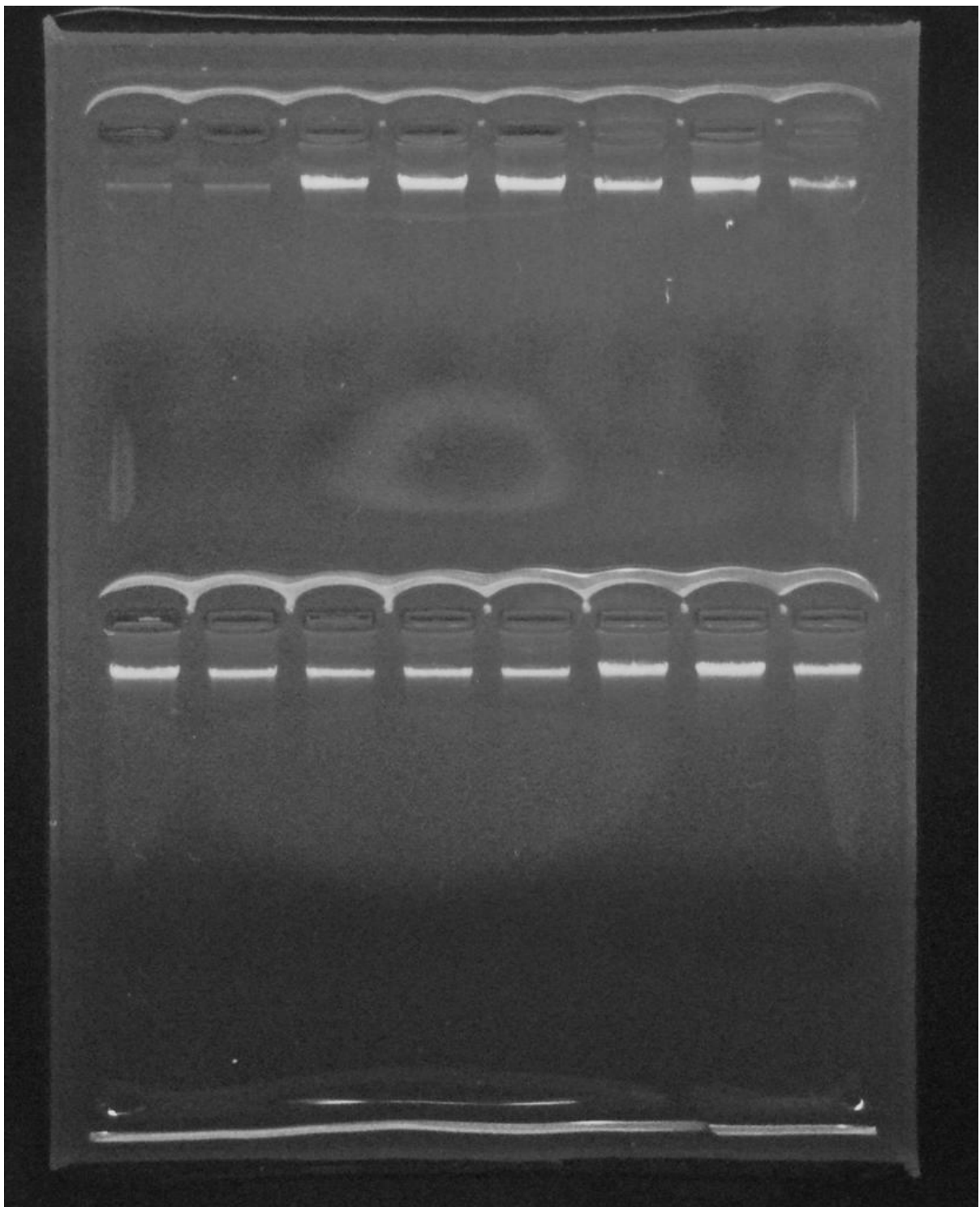

Figure 1

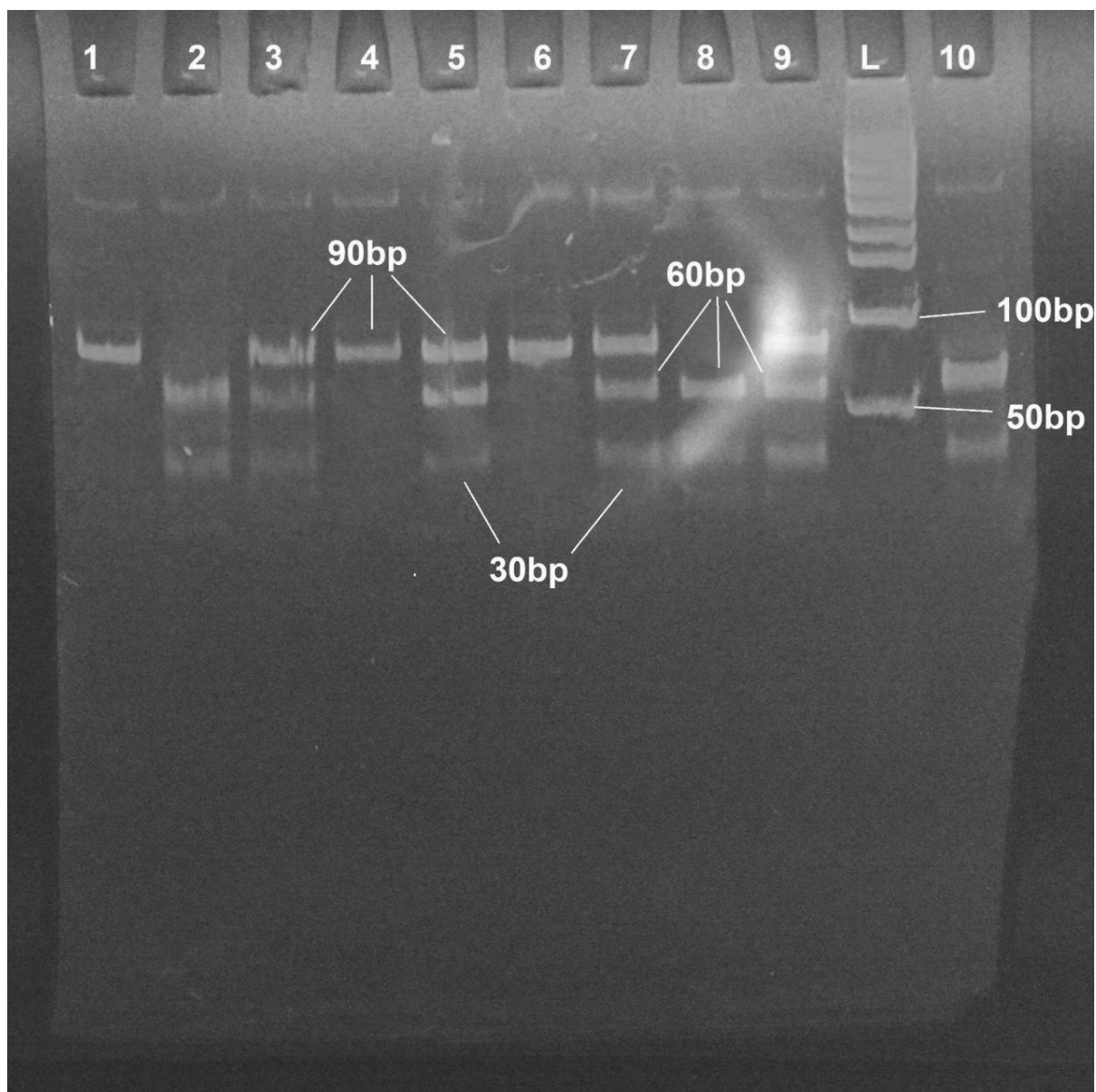

Figure 2

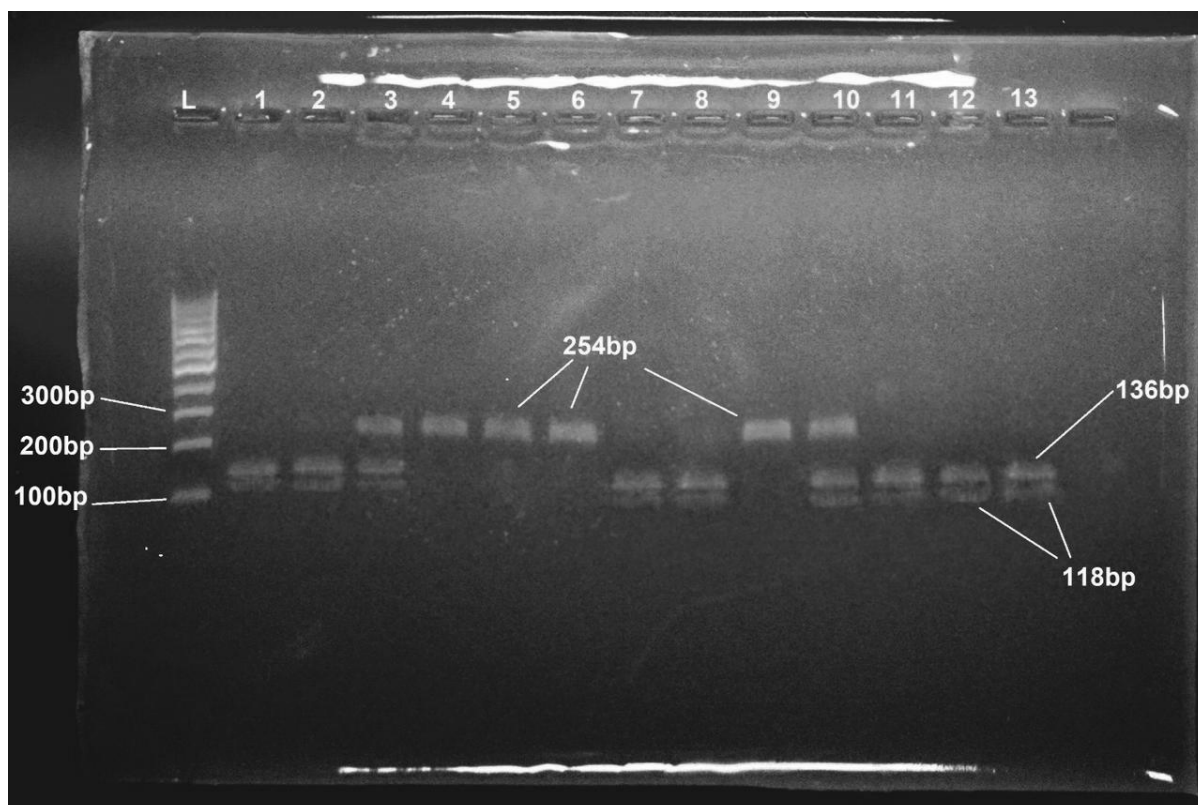

Figure 3

Supplement: Supplementary file 1 — Supplementary Figures. [file 41598_2024_65199_MOESM1_ESM.pdf]
